# The relation between humor styles and nurse burnout: a cross-sectional study in China

**DOI:** 10.3389/fpubh.2024.1414871

**Published:** 2024-11-26

**Authors:** Cuiyun Fang, Shuanghua Fan, Di Chen, Yuan Zhou, Wei Fan

**Affiliations:** ^1^Department of Gastrointestinal Surgery, Liyang People's Hospital, Liyang, China; ^2^Department of Pediatrics, Liyang People's Hospital, Liyang, China

**Keywords:** humor styles, nurse, burnout, emotional exhaustion, cross-sectional study

## Abstract

**Background:**

Nurse burnout is a prevalent issue in healthcare, impacting both nurses’ well-being and patient care quality. This cross-sectional study examined the association between humor styles and nurse burnout.

**Methods:**

A total of 244 nurses in China completed an online self-report measure to assess their humor styles and burnout levels using the Humor Styles Questionnaire (HSQ) and the Maslach Burnout Inventory-Human Services Survey (MBI-HSS). Spearman correlation analysis and stepwise regression analysis were conducted.

**Results:**

The results showed that affiliative and self-enhancing humor were moderately used, while aggressive and self-defeating humor were rated low among the nurses. Emotional exhaustion was moderate, depersonalization was severe, and personal accomplishment was low. Correlation analyses uncovered significant relationships between humor styles and burnout dimensions. Self-enhancing humor exhibited negative correlations with emotional exhaustion and depersonalization, while aggressive and self-defeating humor styles displayed positive correlations with these burnout factors. Affiliative humor was also negatively correlated with depersonalization. Additionally, self-enhancing humor was positively correlated with personal accomplishment, whereas aggressive humor showed negative correlations with this dimension of burnout. Stepwise regression analysis revealed that self-defeating humor positively predicted emotional exhaustion while self-enhancing humor negatively predicted it. Aggressive humor positively predicted depersonalization, and affiliative and self-enhancing humor also positively predicted this dimension of burnout. Self-enhancing humor positively predicted personal accomplishment, while aggressive and self-defeating humor negatively predicted this dimension.

**Conclusion:**

The findings highlighted the importance of considering different types of humor in understanding the various dimensions of nurse burnout. The use of self-defeating and aggressive humor appears detrimental, while the use of self-enhancing humor may be beneficial in mitigating burnout among nurses.

## 1 Introduction

Humor is a complex and multifaceted construct that has been extensively studied in various fields, including psychology, sociology, pedagogy, and medicine (1–4). It is defined as a cognitive and emotional response characterized by amusement and laughter, involving mental processes designed to create and perceive comic stimuli (5, 6). Humor can be understood as any message designed to evoke smiles or laughter, conveyed through action, speech, writing, images, or music (7, 8). The production of humor involves a complex interplay of psychological and cognitive processes, including emotional, cognitive, and value judgments (9). Humor can be classified into various forms, such as jokes, satire, irony, hyperbole, and stereotypes, each with its own unique function and impact (10). While some universal elements of humor exist, the specific expressions and content of humor vary across cultures, often limited by an individual’s cultural background and worldview (11). Several major theories attempt to explain the nature and role of humor, including superiority theory, release theory, incongruity theory, cognitive problems theory, and psychoanalytic perspective (12). Superiority theory suggests that humor stems from feelings of superiority over the weaknesses of others (13), release theory proposes that humor is a release of tension (13), incongruity theory emphasizes the role of inconsistencies between expectations and reality (13), and cognitive-problems theory and psychoanalytic perspectives explain humor in terms of cognitive processing and underlying psychodynamics (12). Additionally, the style of humor is an important aspect of research in this field. One commonly accepted classification is to divide humor into four categories: affiliative humor, which involves using humor to enhance relationships and reduce tension; self-enhancing humor, which involves using humor to cope with stress and maintain a positive outlook; aggressive humor, which involves using humor to enhance one’s status at the expense of others; and self-defeating humor, which involves using humor to gain approval and acceptance from others by putting oneself down (6). Each of these categories is associated with different psychological and behavioral outcomes, and understanding the nuances of each can be critical in workplace settings (14). The use of humor promotes communication, enhances health, helps cope with unpleasant situations, and reduces tension, discomfort, and stress (15). Despite its often positive and adaptive nature, not all humor is beneficial. Affiliative and self-enhancing humor are considered positive humor styles that can promote health, improve social skills, and increase work engagement and well-being (16). Conversely, aggressive and self-defeating humor are considered negative styles, negatively correlated with mental health, and can increase emotional exhaustion and decrease resilience and social competence (17, 18).

Nurse burnout is a persistent and pervasive issue characterized by physical, emotional, and mental exhaustion resulting from prolonged exposure to demanding work environments (19). Current research reveals a concerning prevalence of burnout among nurses, with numerous contributing factors including heavy workloads, understaffing, lack of autonomy, and the emotional strain of caring for patients in distress (20). Additionally, the nature of nursing work, which involves managing high-stress situations and often witnessing suffering and loss, further exacerbates the risk of burnout (21). High levels of burnout symptoms early in a nurse’s life are significantly associated with more frequent symptoms of cognitive dysfunction, depression, and impaired sleep later in life (22). Nurses also face high emotional demands from caring for patients, dealing with patient deaths, and managing complex patient needs, all of which can deplete emotional resources (23). Other factors, such as lack of support from management, role ambiguity, and work-life imbalance, have also been linked to increased burnout among nurses (24, 25). The consequences of nurse burnout are significant, both for individual nurses and the healthcare system as a whole. Burnout has been associated with decreased job satisfaction, increased turnover intentions, and higher rates of absenteeism (26). Burnout can also negatively impact patient care, with studies showing links between nurse burnout and poorer patient outcomes, including increased medical errors and lower patient satisfaction (27, 28). Nurse burnout is a global issue, with high rates reported worldwide. Estimates suggest that up to 30–50% of nurses in the United States experience burnout (29, 30). Similarly, elevated burnout levels have been documented in Europe, with over 25% of nurses in Germany, France, and England reporting high emotional exhaustion (31). High burnout rates, exceeding 30%, have also been reported among nurses in South Korea (32). In China, a nationwide questionnaire survey revealed a burnout rate of up to 50% of nurses (33). While the exact prevalence varies, the widespread nature of nurse burnout across diverse healthcare settings underscores its global significance as a critical challenge facing the nursing profession.

The extant literature has explored the relationship between nurses’ use of various humor styles and their work-related outcomes. Appropriate use of humor can help nurses alleviate work-related stress, foster trusting relationships with patients, and improve patient satisfaction (34). Furthermore, the judicious application of humor by nurses has been found to enhance their job satisfaction and sense of professional pride, ultimately improving the overall quality of nursing care (35). Studies have shown that affiliative and self-enhancing humor styles were strongly associated with higher levels of well-being, sociability, hope, and life satisfaction, while aggressive humor was linked to low life satisfaction and high nursing stress, and self-defeating humor was associated with better health outcomes among nurses (36). However, little literature has comprehensively examined the correlation between different humor styles and nurse burnout (37). The existing literature has predominantly focused on Western samples, with a paucity of research examining these relationships within the unique cultural context of China, where nurse burnout has been particularly problematic (38, 39). Nurses in China often face additional challenges, such as high patient loads, limited resources, and a hierarchical healthcare system, which may contribute to elevated levels of burnout (39). The current study aims to address these gaps in the literature and provide a more comprehensive understanding of the relationship between humor styles and the multidimensional construct of burnout in the Chinese cultural context. By examining the association between different humor styles (affiliative, self-enhancing, aggressive, and self-defeating) and the three dimensions of burnout (emotional exhaustion, depersonalization, and reduced personal accomplishment), this study seeks to contribute to the existing knowledge on this topic.

## 2 Materials and methods

### 2.1 Design

This cross-sectional study was conducted at a tertiary hospital in China, where hospitals are categorized into three grades based on regular government reviews of their overall healthcare capacity and quality. The higher the hospital grade, the greater its overall capacity. The study aimed to explore the characteristics of humor styles and nurse burnout within a single-center cohort and examine the association between them. Our research hypothesis was that positive humor styles, such as affiliative and self-enhancing humor, would be positively associated with nurse burnout, while negative humor styles, such as aggressive and self-defeating humor, would be negatively associated with nurse burnout.

### 2.2 Participants

The data collection period spanned from June to October 2023, during which an online questionnaire was disseminated to all nursing staff at the hospital through the nursing department. The questionnaire provided a comprehensive overview of the study’s objectives, research methodology, and the requirement for informed consent. The questionnaire was uploaded to an online survey platform named Wen Juan Xing. Before starting the questionnaire, participants were presented with an electronic informed consent form, which explained the purpose of the study, mode of participation, and privacy protection. Participants were required to check “I have read and agree to participate in this study” before proceeding to the formal questionnaire.

### 2.3 Materials

The Humor Styles Questionnaire (HSQ) is a widely used self-report measure to assess an individual’s humor styles. It has been translated into over 30 languages and validated for reliability (6, 40). The HSQ consists of four subscales: affiliative humor, self-enhancing humor, aggressive humor, and self-defeating humor. The affiliative humor subscale measures the use of humor to enhance relationships and reduce tension, while the self-enhancing humor subscale assesses the use of humor to cope with stress and maintain a positive outlook. The aggressive humor subscale measures the use of humor to enhance one’s own status at the expense of others, and the self-defeating humor subscale assesses the use of humor to ingratiate oneself with others at the expense of oneself. The final Chinese version of the HSQ consists of 25 items, and participants rated each item on a 7-point Likert scale, with higher scores indicating a higher degree of humor in a given category. Affiliative humor contains 8 items with scores ranging from 8 to 56, self-enhancing humor contains 5 items with scores ranging from 5 to 35, aggressive humor contains 7 items with scores ranging from 7 to 49, and self-defeating humor contains 5 items with scores ranging from 5 to 35. The scores were grouped as follows: high (affiliative ≥41, self-enhancing ≥26, aggressive ≥35, self-defeating ≥26), moderate (affiliative 25–40, self-enhancing 16–25, aggressive 22–34, self-defeating 16–25), and low (affiliative ≤24, self-enhancing ≤15, aggressive ≤21, self-defeating ≤15).

The Maslach Burnout Inventory (MBI) is a widely recognized and extensively used self-report measure specifically designed to evaluate burnout in the workplace, offering tailored versions to suit different industries (41, 42). In this study, the Maslach Burnout Inventory-Human Services Survey (MBI-HSS) version was selected to assess burnout among nurses. The Chinese version of the MBI-HSS utilized in this study consists of 22 items. The emotional exhaustion dimension reflects the depletion of emotional resources and is scored according to the frequency of feelings experienced. It contains 9 items with scores ranging from 0 to 54; the higher the score, the more severe the emotional exhaustion. Similarly, the depersonalization dimension measures apathy and cynicism toward the patient and is scored by assessing the frequency of negative attitudes and reactions. It contains 5 items with scores ranging from 0 to 30; the higher the score, the greater the degree of depersonalization. The personal accomplishment dimension assesses feelings of inefficiency and lack of accomplishment at work. It contains 8 items with scores ranging from 0 to 48, with lower scores indicating a decreased sense of personal accomplishment and a higher degree of burnout. High burnout was defined as emotional exhaustion score ≥27, depersonalization ≥10, and personal accomplishment ≤33; moderate burnout was emotional exhaustion score of 19–26, depersonalization of 6–9, and personal accomplishment 34–39; and low burnout as emotional exhaustion score ≤18, depersonalization ≤5 and personal accomplishment ≥40.

### 2.4 Data collection

The collected data encompassed three key components: demographic data (including age, gender, work experience, education, technical rank, marital status, and the existence of children), the HSQ, and MBI-HSS.

### 2.5 Statistical analysis

The study employed descriptive statistics to present the demographic characteristics and scale scores of the participants. Cronbach’s alpha coefficient was used to analyze the reliability of the HSQ and MBI-HSS. Harman’s single-factor test was conducted to assess the presence of common method bias. For qualitative variables, frequencies and percentages were reported, while means and standard deviations were used for quantitative variables. Spearman correlation tests were used to estimate the correlations between variables. Stepwise regression analysis was then performed to identify the predictive power of different humor styles across the three dimensions of nurse burnout (emotional exhaustion, depersonalization, and personal accomplishment). Statistical analysis was conducted using the Statistical Product and Service Solutions (SPSS) version 28 (IBM Corp., Armonk, NY, United States). A two-sided test was used for significance testing, with the significance level set at *p* < 0.05.

### 2.6 Ethics statement

In adherence to ethical guidelines, this study followed the principles outlined in the Declaration of Helsinki and received approval from the Ethical Review Committee of Liyang People’s Hospital (No. LY2023034). Informed consent was obtained from all subjects.

## 3 Results

During the study period, a total of 754 nurses were registered at Liyang People’s Hospital, and questionnaires were distributed to all of them. Ultimately, 244 nurses signed the informed consent form and completed the questionnaire items, resulting in a response rate of 32.4%. Among the participants, 242 (99.2%) were female and 2 (0.8%) were male, with ages ranging from 19 to 55 years and a mean age of 31.57 ± 6.57 years. A comprehensive overview of the demographic characteristics is provided in Table 1.

**Table 1:** Demographic information of participants.

Variables	*N* (%)
Work experience (years)	
<5	60 (24.6)
5–10	88 (36.1)
10–15	72 (29.5)
>15	24 (9.8)
Education	
Junior college or below	36 (14.8)
Bachelor	188 (77.0)
Master	20 (8.2)
Technical rank	
Student	12 (4.9)
Junior	92 (37.7)
Intermediate	124 (50.8)
Senior	16 (6.6)
Marital status	
Unmarried	56 (23.0)
Married	184 (75.4)
Others	4 (1.6)
Existence of children	
0	84 (34.4)
1	112 (45.9)
2	44 (18.0)
≥3	4 (1.6)

The Harman single-factor test was used to assess common method bias. Factor analysis was performed on all items of the scales, and the first extracted factor had a variance explanation rate of 36.38%, which is less than 40%, indicating that there was no significant common method bias. In the current study, the Cronbach’s alpha coefficients for the HSQ subscales were 0.87 for affiliative humor, 0.81 for self-enhancing humor, 0.83 for aggressive humor, and 0.78 for self-defeating humor. The Cronbach’s alpha coefficients for the MBI-HSS subscales were 0.89 for emotional exhaustion, 0.81 for depersonalization, and 0.87 for personal accomplishment, indicating good reliability of the instruments. The scores of HSQ and MBI-HSS are summarized in Table 2. Analysis of the HSQ revealed that affiliative and self-enhancing humor were rated as moderate, while aggressive and self-defeating humor were rated as low. In the MBI-HSS, emotional exhaustion was rated as moderate, depersonalization as high, and personal accomplishment as low.

**Table 2:** Scores of participants in HSQ and MBI-HSS.

Variables	Mean ± SD (min, max)
HSQ	
Affiliative humor	28.48 ± 6.65 (8, 48)
Self-enhancing humor	23.16 ± 7.78 (5, 35)
Aggressive humor	12.89 ± 8.26 (7, 41)
Self-defeating humor	10.03 ± 6.56 (5, 31)
MBI-HSS	
Emotional exhaustion	25.84 ± 12.71 (3, 54)
Depersonalization	13.38 ± 4.87 (5, 31)
Personal accomplishment	29.08 ± 9.05 (8, 48)

SD, standard deviation; HSQ, Humor Styles Questionnaire; MBI-HSS, Maslach Burnout Inventory-Human Services Survey.

Correlation analysis revealed significant relationships between different humor styles and the three dimensions of the MBI-HSS, as outlined in Table 3. Self-enhancing humor style had significant negative correlations with emotional exhaustion and depersonalization, while aggressive and self-defeating humor styles displayed positive correlations with emotional exhaustion and depersonalization. Affiliative humor was also found to have a negative correlation with depersonalization. Additionally, self-enhancing humor exhibited a positive correlation with personal accomplishment, while aggressive humor showed a negative correlation with personal accomplishment.

**Table 3:** Correlational analysis of humor styles and nurse burnout.

	Affiliative humor	Self-enhancing humor	Aggressive humor	Self-defeating humor
Emotional exhaustion	0.071	−0.272^*^	0.291^*^	0.312^*^
Depersonalization	−0.570^**^	−0.258^*^	0.595^*^	0.533^*^
Personal accomplishment	0.095	0.271^*^	−0.169^*^	0.004

*p < 0.01.

The results of the stepwise regression analysis presented in Table 4 provided insights into the relationship between different types of humor and the three dimensions of nurse burnout. For emotional exhaustion, the model indicated that self-defeating humor positively predicted emotional exhaustion (*β* = 0.33, *p* < 0.01), while self-enhancing humor negatively predicted emotional exhaustion (*β* = −0.28, *p* < 0.01). Regarding depersonalization, the model showed that aggressive humor positively predicted depersonalization (*β* = 0.65, *p* < 0.01), while affiliative humor (*β* = 0.28, *p* < 0.01) and self-enhancing humor (*β* = 0.23, *p* < 0.01) also positively predicted this dimension of burnout. For personal accomplishment, the model revealed that self-enhancing humor positively predicted personal accomplishment (*β* = 0.22, *p* < 0.01), while aggressive humor (*β* = −0.64, *p* < 0.01) and self-defeating humor (*β* = 0.50, *p* < 0.01) negatively predicted this dimension.

**Table 4:** Stepwise regression analysis results in predicting the three dimensions of nurse burnout.

	Variables	*R* ^2^	*F*	*B*	β
Emotional exhaustion	Self-defeating humor	0.19	28.52*	0.65	0.33*
	Self-enhancing humor			−0.47	−0.28*
Depersonalization	Aggressive humor	0.81	343.60*	0.38	0.65*
	Affiliative humor			0.21	0.28*
	Self-enhancing humor			0.14	0.23*
Personal accomplishment	Self-enhancing humor	0.13	12.18*	0.26	0.22*
	Aggressive humor			−0.70	−0.64*
	Self-defeating humor			0.69	0.50*

*p < 0.01.

## 4 Discussion

The present study investigated the correlation between humor styles and nurse burnout among nurses at Liyang People’s Hospital. The response rate of 32.4% indicated a substantial level of participation. The predominance of female nurses in the sample aligns with the gender distribution commonly observed in Chinese nurses (43). Analysis of humor styles using the HSQ indicated moderate scores for affiliative and self-enhancing humor, and low scores for aggressive and self-defeating humor, suggesting a predominance of positive and affiliative humor styles among the nurses. Furthermore, the assessment of burnout levels using the MBI-HSS revealed moderate levels of emotional exhaustion, high levels of depersonalization, and low levels of personal accomplishment. The correlation analysis demonstrated significant relationships between humor styles and burnout dimensions, with specific humor styles showing significant correlations with emotional exhaustion, depersonalization, and personal accomplishment. The results of the stepwise regression analysis suggested that the use of self-defeating humor positively predicted emotional exhaustion while self-enhancing humor negatively predicted it. Regarding depersonalization, the model showed that aggressive, affiliative, and self-enhancing humor positively predicted this dimension of burnout. For personal accomplishment, the model revealed that self-enhancing humor positively predicted this dimension, while aggressive and self-defeating humor negatively predicted it. Overall, the results highlighted the importance of considering different types of humor in understanding the various dimensions of nurse burnout. The use of self-defeating and aggressive humor appears to be detrimental, while the use of self-enhancing humor may be beneficial in mitigating burnout among nurses.

The moderate scores for affiliative and self-enhancing humor styles among the nurses in this study are indicative of a tendency to use humor to foster positive relationships and cope with stress. This aligns with previous research suggesting that affiliative humor can contribute to a supportive and cohesive work environment, potentially serving as a protective factor against burnout (44, 45). Similarly, the moderate prevalence of self-enhancing humor, involving the use of humor to maintain a lighthearted perspective and cope with stress, reflects the nurses’ adaptive coping strategies. These findings are consistent with studies indicating that self-enhancing humor can act as a resilience factor, helping individuals maintain a positive outlook in challenging situations (46). The low scores for aggressive and self-defeating humor styles further indicate a minimal inclination among nurses to use humor in ways that may be detrimental to themselves or others. Overall, the prevalence of positive humor styles suggests that nurses at Liyang People’s Hospital utilize humor as a constructive coping mechanism, potentially influencing their experiences of burnout.

The assessment of nurse burnout using the MBI-HSS revealed noteworthy findings regarding the emotional exhaustion, depersonalization, and personal accomplishment dimensions. The moderate levels of emotional exhaustion observed among the nurses in this study indicate a significant degree of work-related stress and depletion of emotional resources. This finding is particularly concerning, as emotional exhaustion is a core component of burnout and can have detrimental effects on nurses’ well-being and patient care (47–49). The high levels of depersonalization, characterized by negative and cynical attitudes toward patients, suggest a potential erosion of empathy and compassion among nurses (50). This is a critical issue, as depersonalization can lead to decreased quality of patient care and further contribute to the cycle of burnout (51). Moreover, the low levels of personal accomplishment highlight a diminished sense of professional efficacy and achievement among the nurses, which can impact their motivation and job satisfaction (52). These findings underscore the multifaceted nature of nurse burnout and emphasize the need for targeted interventions to address emotional exhaustion, depersonalization, and reduced personal accomplishment among nurses.

Based on the study’s findings, it’s clear there is a significant association between humor styles and nurse burnout. The results revealed that nurses who exhibit higher self-enhancing and affiliative humor styles tend to report lower levels of emotional exhaustion and depersonalization, while also demonstrating higher levels of personal accomplishment. Conversely, nurses who exhibited higher aggressive and self-defeating humor styles tend to experience higher levels of emotional exhaustion and depersonalization. These findings underscore the correlation between humor styles and nurse burnout. This supports existing literature suggesting that affiliative and self-enhancing humor can serve as adaptive coping mechanisms, buffering against the negative effects of job-related stress and burnout (53). On the other hand, nurses who exhibited higher aggressive and self-defeating humor styles were associated with higher levels of emotional exhaustion and depersonalization. The implications of these findings are substantial for healthcare organizations and nursing management. Interventions aimed at promoting positive humor styles among nurses could serve as a preventive strategy for addressing and reducing burnout (54). This may involve incorporating humor-based training programs or workshops to cultivate positive humor styles and enhance coping mechanisms for stress and emotional exhaustion (55). By fostering a workplace culture that values and encourages positive humor, healthcare organizations can strive to create a supportive environment that promotes nurse well-being and, in turn, improves the quality of patient care (56). In conclusion, the study’s findings highlight the significant association between humor style and nurse burnout, emphasizing the potential for targeted interventions to harness the positive impact of humor on mitigating burnout and enhancing the well-being of nurses.

While this study provides valuable insights into the association between humor styles and nurse burnout, several limitations should be acknowledged. Firstly, the cross-sectional design of the study limits the ability to establish causality or temporal relationships between humor styles and nurse burnout dimensions. Longitudinal research would provide a more comprehensive understanding of how humor styles may influence the development and progression of nurse burnout over time. Additionally, the use of self-report measures for both humor styles and burnout may introduce common method bias and social desirability effects, potentially impacting the accuracy of the reported relationships. Future research could benefit from incorporating multiple data sources, such as observational or peer ratings of humor styles, and objective measures of burnout. Furthermore, the study sample was drawn from a specific geographic region and healthcare setting, potentially limiting the generalizability of the findings to other nursing populations. Replication of the study with diverse samples would enhance the external validity of the results.

## 5 Conclusion

In conclusion, this study provides valuable insights into the complex relationships between different types of humor and the multifaceted nature of nurse burnout. The findings highlight the importance of promoting the use of self-enhancing humor among nurses. Conversely, the study suggests that the reliance on self-defeating and aggressive forms of humor may be detrimental. These results underscore the importance of considering the nuances of humor styles when addressing the issue of burnout in the nursing profession.

## Data Availability

The original contributions presented in the study are included in the article/supplementary material, further inquiries can be directed to the corresponding author.
